# Altered properties and structures of root exudate polysaccharides in a root hairless mutant of barley

**DOI:** 10.1093/plphys/kiac341

**Published:** 2022-07-25

**Authors:** Andrew F Galloway, Jumana Akhtar, Emma Burak, Susan E Marcus, Katie J Field, Ian C Dodd, Paul Knox

**Affiliations:** Centre for Plant Sciences, Faculty of Biological Sciences, University of Leeds, Leeds LS2 9JT, UK; Centre for Plant Sciences, Faculty of Biological Sciences, University of Leeds, Leeds LS2 9JT, UK; Lancaster Environment Centre, Lancaster University, Lancaster LA1 4YQ, UK; Centre for Plant Sciences, Faculty of Biological Sciences, University of Leeds, Leeds LS2 9JT, UK; Plants, Photosynthesis and Soil, School of Biosciences, University of Sheffield, Sheffield S10 2TN, UK; Lancaster Environment Centre, Lancaster University, Lancaster LA1 4YQ, UK; Centre for Plant Sciences, Faculty of Biological Sciences, University of Leeds, Leeds LS2 9JT, UK

## Abstract

Root exudates and rhizosheaths of attached soil are important features of growing roots. To elucidate factors involved in rhizosheath formation, wild-type (WT) barley (*Hordeum vulgare* L. cv. Pallas) and a root hairless mutant, *bald root barley* (*brb*), were investigated with a combination of physiological, biochemical, and immunochemical assays. When grown in soil, WT barley roots bound ∼5-fold more soil than *brb* per unit root length. High molecular weight (HMW) polysaccharide exudates of *brb* roots had less soil-binding capacity than those of WT root exudates. Carbohydrate and glycan monoclonal antibody analyses of HMW polysaccharide exudates indicated differing glycan profiles. Relative to WT plants, root exudates of *brb* had reduced signals for arabinogalactan-protein (AGP), extensin, and heteroxylan epitopes. In contrast, the root exudate of 2-week-old *brb* plants contained ∼25-fold more detectable xyloglucan epitope relative to WT. Root system immunoprints confirmed the higher levels of release of the xyloglucan epitope from *brb* root apices and root axes relative to WT. Epitope detection with anion-exchange chromatography indicated that the increased detection of xyloglucan in *brb* exudates was due to enhanced abundance of a neutral polymer. Conversely, *brb* root exudates contained decreased amounts of an acidic polymer, with soil-binding properties, containing the xyloglucan epitope and glycoprotein and heteroxylan epitopes relative to WT. We, therefore, propose that, in addition to physically structuring soil particles, root hairs facilitate rhizosheath formation by releasing a soil-binding polysaccharide complex.

## Teaser

The root exudate of a root hairless mutant of barley, relative to wild type, has an altered pattern of polysaccharide epitopes and lesser amounts of an acidic soil-binding polysaccharide complex.

## Introduction

The interactions of plant roots with the soil around them are a major feature of plant growth and functioning. These complex interactions can be viewed as components of root phenotypes that extend beyond the surface of outer root cells (de la Fuente Canto et al., 2020). They are mediated by a diversity of plastic growth processes and secreted molecular factors that chemically and biologically influence the surrounding soil to create the rhizosphere with altered soil properties. In most species, roots become enveloped by a layer of soil as they grow, known as a rhizosheath, which remains attached to roots upon excavation (Brown et al., 2017; Pang et al., 2017; Ndour et al., 2020). Rhizosheath formation may help plants sustain resource (water and nutrients) uptake, as rhizosheath soil has a higher moisture content than the surrounding bulk soil (Young, 1995; Basirat et al., 2019) and rhizosheath mass has been associated with phosphate uptake (George et al., 2014). Several species of semi-arid savanna grasses and maize (*Zea mays*) increase their rhizosheath thickness during drought, which may assist water uptake (Watt et al., 1994; Hartnett et al., 2013; Basirat et al., 2019). Several studies have documented rhizosheath-specific microbiomes (Dennis et al., 2010; Marasco et al., 2018; Ndour et al., 2020) that may also be important for root functions. Since these studies have highlighted a range of potential functions for rhizosheaths, it is important to understand the mechanisms contributing to their formation and stabilization.

Root hairs and adhesive polymers have long been implicated in the formation of rhizosheaths. However, due to the technical challenges of elucidating these factors, specifically the analysis of root exudates, the inter-relations of root hairs and their secretions and associated mechanisms remain unclear (Watt et al., 1993; Koebernick et al., 2017; Holz et al., 2018; Ndour et al., 2020). Genotypes with root hairs can bind more soil to the roots, while mutants lacking root hairs show limited rhizosheath development (Haling et al., 2013; George et al., 2014; Burak et al., 2021). Moreover, while root hair length is strongly positively correlated with rhizosheath weight in wheat (*Triticum aestivum*; Delhaize et al., 2012), weaker relationships are detected in other species such as barley (*Hordeum vulgare*; George et al., 2014). Limited rhizosheath formation (rather than a complete absence of the rhizosheath) even in root hairless mutants suggests that adhesive factors can also be released from, and/or presented at the surface of, nonroot hair cells (Burak et al., 2021).

The strength with which rhizosheath soil is bound to roots also varies among species, with rhizosheaths more easily removed from some species than others (Brown et al., 2017). The nature of adhesive factors and the mechanisms of rhizosheath stabilization remain unclear. Recent work has begun to address these aspects of root physiology. Novel assays are being used to dissect the adhesiveness of root hairs using genetics in the Arabidopsis (*Arabidopsis thaliana*) model system (De Baets et al., 2020; Eldridge et al., 2021) and antibody-based methods are being developed to track the release from roots of polysaccharides with soil-binding properties (Galloway et al., 2018, 2020). Extensive work has focused on the high molecular weight (HMW) polymers appearing at root apices in the form of root tip mucilage but less attention has been given to HMW factors that may be released along the root axes to influence interactions with soil and root processes (see Brown et al., 2017; Galloway et al., 2018, 2020). In wheat and maize root exudates, polysaccharides >30,000 Da have been collected from hydroponic systems and soil-binding carbohydrate components analyzed (Galloway et al., 2020). Tracking of glycan epitopes (including those of arabinogalactan-proteins (AGPs), heteroxylan, and xyloglucan), in these preparations with relevant monoclonal antibodies (MAbs) has indicated exudate release along root axes, at root hair surfaces and on soil particles (Galloway et al., 2020). Xyloglucan, a major polysaccharide of eudicot and noncommelinid cell walls has been discovered to be released by a wide range of land plants (including cereals) and to have strong soil-binding capacities (Akhtar et al., 2018; Galloway et al., 2018). Additionally, xyloglucan has been indicated to be a component of the complex branched polysaccharides released by wheat and maize roots (Galloway et al., 2020).

To explore the importance of physical (root hairs) and chemical (root exudation of polysaccharides) factors in influencing rhizosheath development, we studied wild-type (WT) barley (*Hordeum vulgare* L. cv. Pallas) and its root hairless mutant *bald root barley* (*brb*; Gahoonia et al., 2001). Previous research has utilized this mutant to study rhizosheath formation, root carbon efflux, nutrient uptake, plant growth, and transpiration (e.g. Gahoonia et al., 2001; Dodd and Diatloff, 2016; Pausch et al., 2016; Carminati et al., 2017; Holz et al., 2018; Burak et al., 2021). Here we assess the capacity of the two genotypes to form rhizosheaths, in conjunction with carbohydrate analyses and glycan antibody-based analyses of released and root-surface polysaccharides. These analyses have provided insights into the root hair contributions to the range and dynamics of barley root exudate polysaccharides and associated soil-binding factors.

## Results

### Root hairs enhance rhizosheath formation in barley

**Figure 1:**
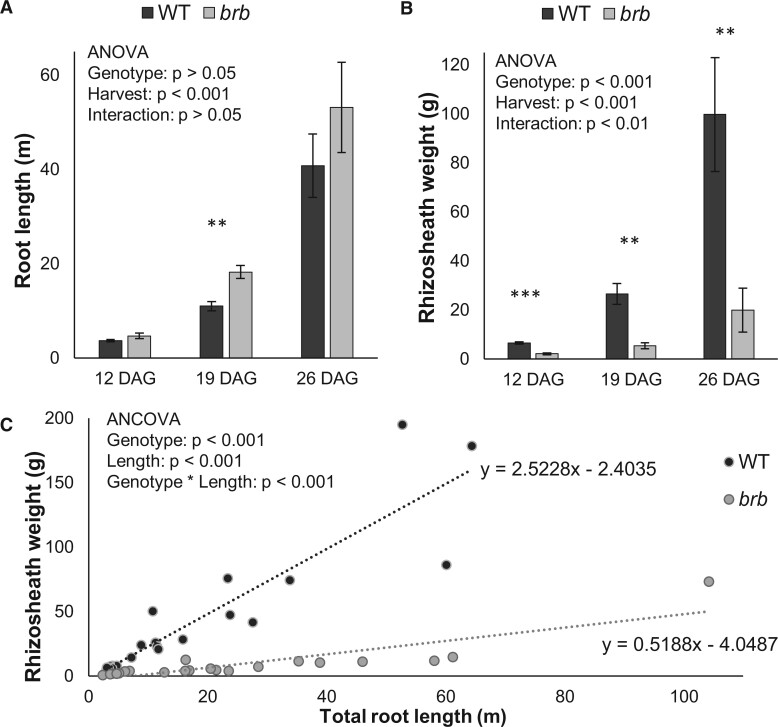
Impact of root hairs on rhizosheaths. Barley plants were grown in a well-watered clay loam and roots were harvested over three consecutive weeks; 12, 19, and 26 DAG. Root length (A) and rhizosheath weight (B) of root hairless *brb* and WT barley plotted against each other (C); standard error bars in (A) and (B) are means of 7 biological replicates with significant differences between genotypes denoted: **P < 0.01, ***P < 0.001. Each point in (C) is an individual plant. Although *brb* tends to produce more root length (A), WT barley consistently binds more soil to its roots (B). Comparing the slopes of the regression lines in (C) indicates that WT barley can bind 4.8 times more soil per unit of root length than the root hairless *brb* mutant.

To confirm the role of root hairs in rhizosheath formation, soil retention by the roots of WT barley and the root hairless mutant *brb* was quantified 3 times up to 26 d after germination (DAG). Generally, total root length of *brb* was higher (by 40% averaged over the experiment) than for WT, with significantly (*P* < 0.01) longer roots after 19 d of growth (Figure 1A). Despite its smaller root length, WT barley bound significantly (∼4-fold, *P* < 0.001) more soil at each harvest than *brb* (Figure 1B). When accounting for the discrepancy in root lengths, WT barley bound 4.8-fold more soil per unit of root length than *brb* (Figure 1C); as indicated by a highly significant (*P* < 0.001) genotype*root length interaction. Thus, root hairs are required for maximal rhizosheath formation since the root hairless mutant *brb* had a limited rhizosheath as previously reported (Burak et al., 2021).

### HMW root exudates of WT and *brb* plants have differing capacities for soil-binding

**Figure 2:**
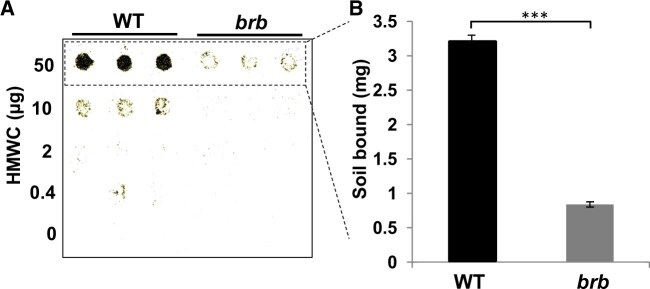
HMW root exudates from WT barley have greater soil-binding capacity than those from *brb* exudates*.* Defined amounts of barley WT and *brb* HMW (>30 KDa) root exudates were applied in 5 µL spots onto nitrocellulose sheets and soil-binding capacity was determined. A, Representative soil adhesion blot, with three replicates per sample. B, Quantification of weight of soil-bound at the 50 µg spots. *n* = 3, ±SD, *P* < 0.001 (*t* test) indicated by three asterisks.

To study the role of HMW root exudates in rhizosheath formation, WT and *brb* plants were grown in a hydroponic system and HMW (>30,000 Da) compounds were collected and their soil-binding properties were determined as described previously (Akhtar et al., 2018; Galloway et al., 2020). Using a nitrocellulose-based soil-binding assay and expressed on a per weight basis, the *brb* HMW exudate bound <1 mg soil/µg exudate whereas the WT exudate bound over 3-fold more (>3 mg soil/µg exudate) as shown in Figure 2.

### Carbohydrate and glycan epitope analysis of WT and *brb* root exudate polysaccharides

**Figure 3:**
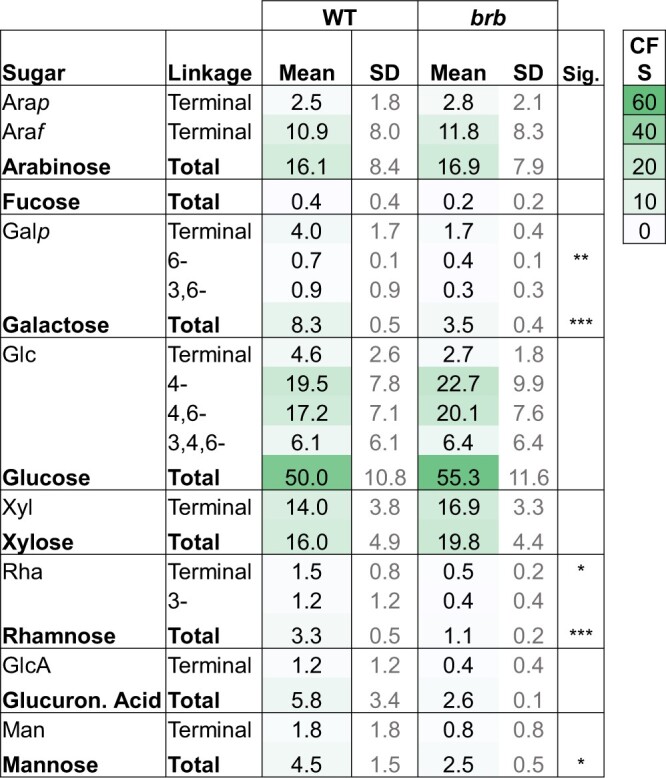
Monosaccharide linkage analysis of root exudates of WT and *brb* plants. Only most abundant sugars/linkages are shown with full analysis in Supplementary information. Data expressed as Mol % and as means of four analyses alongside SD. Significance differences (Sig.) using *t* test in the means between the genotypes are indicated **P < 0.05, ***P < 0.001. Comparative formatting with green shading is shown for mean values only including for linkages within each monosaccharide total. A comparative formatting scale (CFS) of shading from mol % values is also shown.

Monosaccharide linkage analysis of the HMW compounds equivalent to those used in the soil-binding assays indicated differences between WT and *brb* in released polysaccharides, as shown in Figure 3 (major residues detected) and Supplemental Table S1 (full analysis). Both exudates contained a wide range of linkages as previously reported for wheat and maize root exudates collected hydroponically (Galloway et al., 2020). Notable features of the analyses of both WT and *brb* barley genotypes are that over 50% of all glycosyl residues are glucosyl (with a great diversity of linkages, Supplemental Table S1) and that in the region of 40% of all linkages are terminal, indicative of extensive branching of the exudate polysaccharides. WT exudates had a significantly (*P* < 0.001) higher proportion of galactosyl, rhamnosyl, and mannosyl residues than *brb* exudates (Figure 3). Despite high variation between samples, *brb* exudates tended to have increased xylosyl residues. Thus, monosaccharide linkage analysis demonstrated quantitative and qualitative differences in root exudation between the two genotypes.

**Figure 4:**
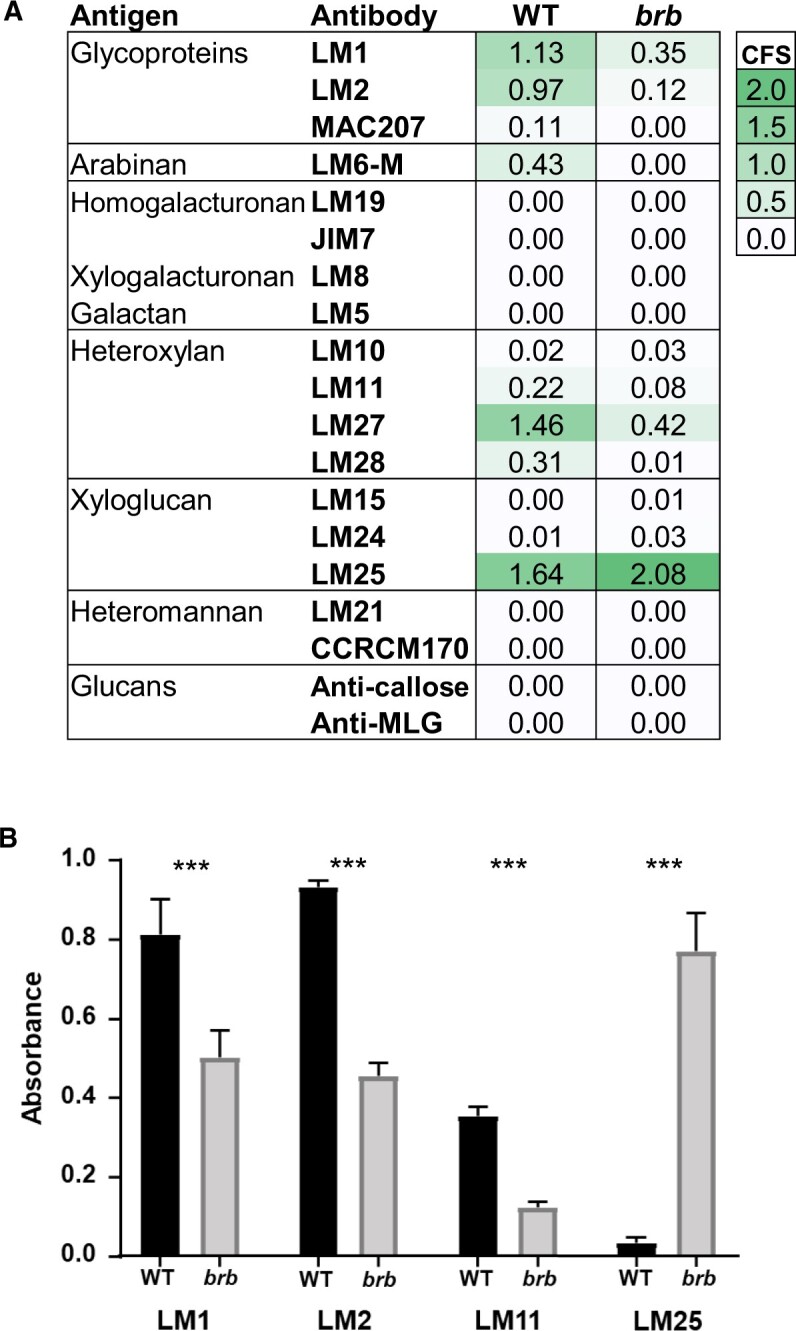
Glycan epitope mapping of barley WT and *brb* HMW root exudates. A, ELISA data for plant glycan MAbs against WT and *brb* root HMW exudates, collected hydroponically, coated onto microtiter plates at 10 µg/mL. Data shown are means of three replicates. SD in all cases less than 0.1 au units. Comparative formatting with green shading is shown alongside a comparative formatting scale (CFS) of shading from 0 to 2 absorbance units. B, Four MAbs used to directly screen unprocessed hydroponates. To get absorbance values in equivalent range for each antibody the hydroponates were diluted 125-fold for LM2 and LM11, 625-fold for LM1, and 3,125-fold for LM25. *n* = 3, ±SD, *P* < 0.001.

To further explore exudate polysaccharides, the presence of glycan epitopes in the root exudates was determined by screening with a selection of MAbs (informed by previous work with cereal root exudates and the presence of the glycosyl linkages as determined by the carbohydrate analysis). Initial screening by ELISA indicated that when immobilized on ELISA plates at 10 µg/mL, the HMW exudate materials contained epitopes for glycoproteins (AGP and extensin), heteroxylan, and xyloglucan (Figure 4A). Noted absences were epitopes for pectin, glucans, and heteromannan. The LM6-M 1,5-arabinan epitope which can be associated with both rhamnogalacturonan-I pectin and AGPs was detected in WT but not *brb* exudates. In the absence of any other pectic epitopes, its presence is suggestive of an AGP antigen. All epitopes detected in WT exudate had relatively reduced signals in *brb* per unit weight of exudate (at least less than one-third of the WT signal), apart from the LM25 xyloglucan epitope which was greatly elevated in *brb* exudates relative to WT. To explore this further using appropriate dilutions of unprocessed hydroponates, the occurrence of representative epitopes of extensin and AGP glycoproteins, heteroxylan, and xyloglucan was determined in the linear range of ELISA response (<1.0 absorbance units) as shown in Figure 4B. As with the processed hydroponates, this analysis indicated that the glycoprotein/heteroxylan epitopes were significantly (*P* < 0.001) decreased by 40%–60% in *brb* exudates relative to wild type. In contrast, the LM25 xyloglucan epitope was detected at a 25-fold higher level in the *brb* exudate relative to WT (*P* < 0.001) as shown in Figure 4B. Thus, glycan epitope mapping showed qualitative and quantitative differences between WT and *brb* root exudates.

### Exudate glycan epitopes are released from root axes of WT and *brb* barley seedlings

**Figure 5:**
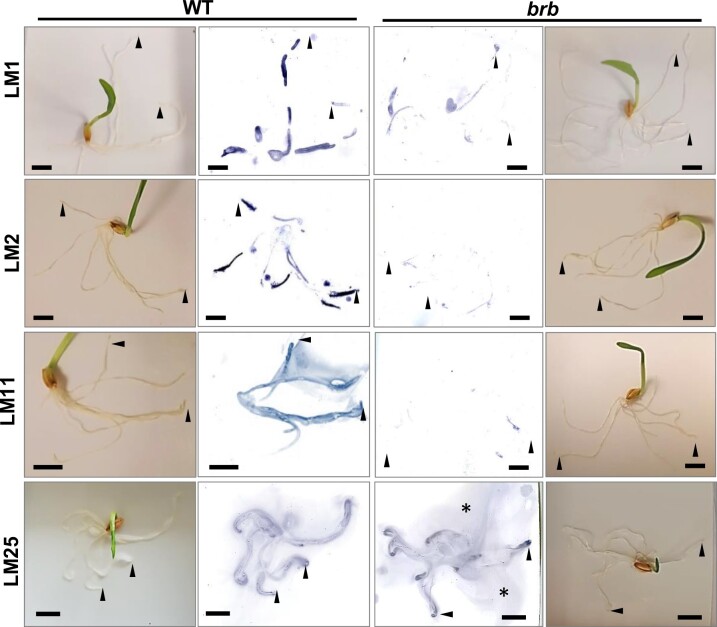
Barley root prints on nitrocellulose to monitor the release of glycan epitopes in WT and *brb* seedlings. Seedlings were placed on nitrocellulose sheets for 2 h, removed and sheets then probed with MAbs LM1 extensin, LM2 AGP, LM11 xylan, and LM25 xyloglucan. Paired images of seedlings in situ and developed nitrocellulose sheets. The LM1, LM2, and LM11 epitopes were less abundant on *brb* seedling sheets and the LM25 epitope appeared more abundant in smears (asterisks) and at root apices (arrowheads), relative to WT. Prints representative of at least three replicates. Bars = 10 mm.

As glycan epitopes are present in root exudates, we explored their release from young seedling root axes in short-duration experiments by placing young seedlings on moist nitrocellulose sheets for 2 h. The seedlings were then removed and sheets probed with MAbs using standard immunochemical procedures. Representative seedling blots (Figure 5) showed that the LM1 extensin, LM2 AGP, and LM11 xylan epitopes were released along root axes but much less so from *brb* seedlings. However, in contrast, the LM25 xyloglucan epitope did not show a reduction of signal for the *brb* mutant relative to WT. There was an indication of a possible enrichment of the LM25 xyloglucan epitope release from root apices, and a smearing of the LM25 antigen across the sheet suggesting a highly soluble polymer. These observations confirm and extend the differential response observed in the ELISA analyses of exudates between the glycoprotein/heteroxylan epitopes and the xyloglucan epitope in the WT/*brb* root exudates.

### Release of the LM25 xyloglucan epitope from root axes and root apices

**Figure 6:**
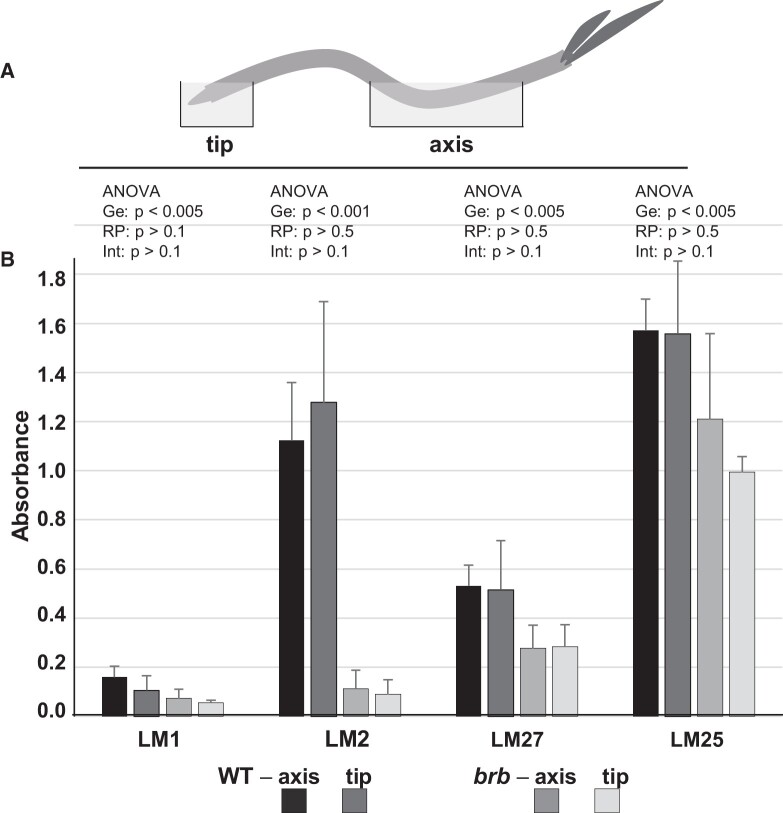
Barley root tip and root axis incubations and immunoprofiling of WT and *brb* seedlings. A, Schematic diagram of seedling incubation showing incubation of root tip and root axis regions in water. Seedlings (7- to 8-d-old) were placed in a humid chamber and 0.5–1 cm of root tip incubated in 1.5 mL water and a region of root body (3–5 cm) separated from root tip by 2 cm was incubated in 5.5 mL water for 2 h. B, ELISA results of 5-fold dilutions of collected incubation samples with LM1 extensin, LM2 AGP, LM27 heteroxylan, and LM25 xyloglucan MAbs. *n* = 5, ±SD, Statistical analysis (two-factor with replication ANOVA, Ge = genotype, RP = root position, Int = interaction) shown above for each MAb.

**Figure 7:**
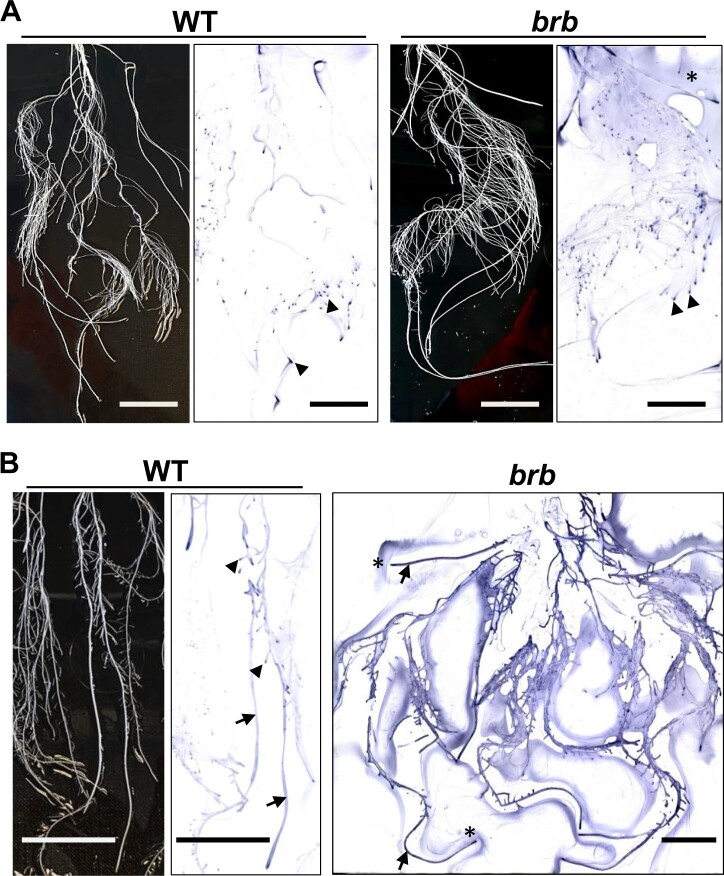
Barley root systems and immunoprints on nitrocellulose sheets developed with the LM25 xyloglucan MAb. WT and *brb* plants grown in hydroponic systems for between 14 and 20 d. A, Root systems positioned on Perspex trays and overlain with nitrocellulose sheets for 10 min before removal and processing of sheets. B, Root systems positioned on Perspex trays, overlain with nitrocellulose, then root systems/nitrocellulose inverted so that roots rested on nitrocellulose for 30 min. WT paired images show regions of root system/print with varying patterns of the LM25 epitope occurrence along root axes (arrows) and root tips (arrowheads). Equivalent *brb* immunoprint shows extensive LM25 epitope signal at all root surfaces and also in soluble exudate around roots (asterisks). Prints representative of at least three replicates. Bars = 30 mm.

To explore further the potential release of the LM25 xyloglucan epitope from both root tips and root axes, a series of incubation and immunoprinting experiments were carried out. Young (7- to 8-d-old) seedlings of both genotypes were incubated so that tip regions and a region of the root axis back from root tip were incubated separately in water for 2 h (see schematic Figure 6A) and the water samples were then analyzed for several exudate epitopes including the LM25 xyloglucan epitope as shown in Figure 6. The ELISA of 10-fold dilutions indicate that the LM1 extensin, LM2 AGP, LM27 heteroxylan, and LM25 xyloglucan epitopes were readily detected in both root tip and root axis samples indicating their release from root axes (Figure 6B). In all cases, relative epitope detection was less in *brb* samples relative to WT, with this differential being most clear for the LM2 AGP epitope and least different for the LM25 xyloglucan epitope. To explore xyloglucan release further in more developed root systems, whole roots (that had grown for ∼2 weeks in hydroponic systems) were immunoprinted. Short-term direct printing (10 min, nitrocellulose sheets above) of arranged root systems indicated that the LM25 xyloglucan epitope was particularly abundant at root tips (Figure 7A). Longer term (30 min) incubations with roots resting on nitrocellulose sheets indicated that the LM25 epitope was abundantly released from along the root axes of *brb* root (relative to WT) and often present in soluble material away from the roots (Figure 7B). Together, these observations suggest highly variable release of xyloglucan from both root tips and root axes and a greatly enhanced increase in the release of the LM25 xyloglucan epitope from *brb* roots relative to WT roots.

### Immunolabelling of intact roots demonstrates exudate epitope occurrence at root hairs

**Figure 8:**
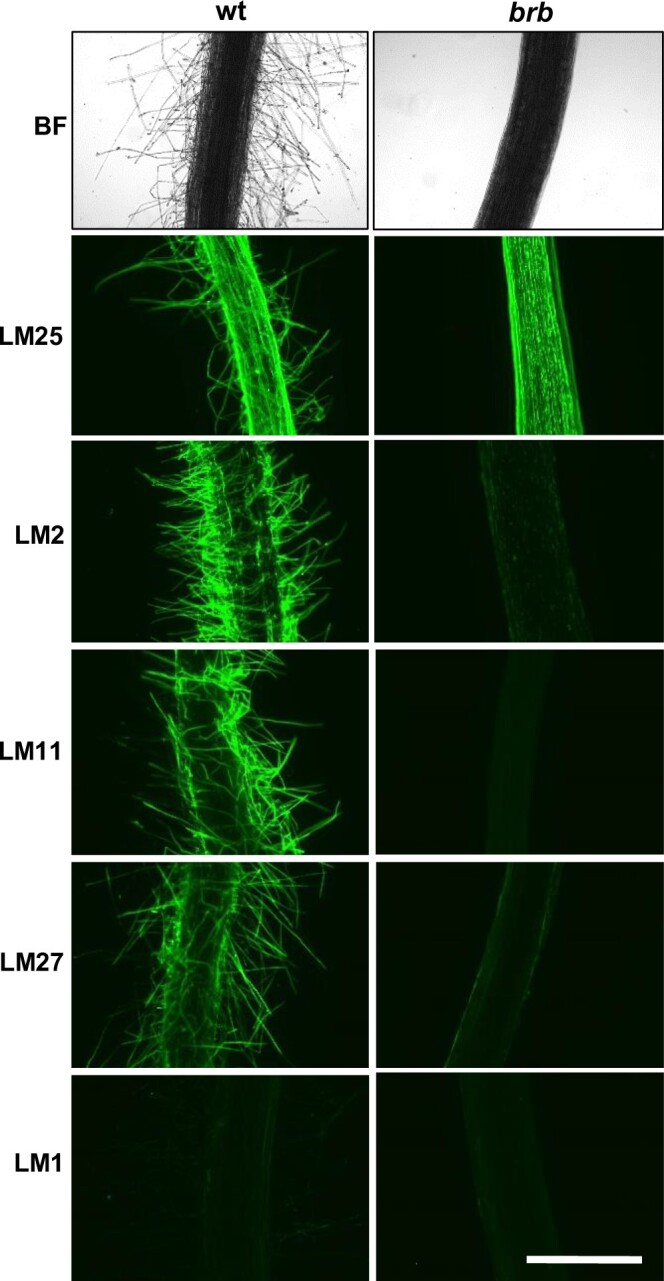
Whole-mount immunofluorescence analysis of root exudate glycan epitopes at barley root surfaces. Paired micrographs of WT and root hairless *brb* roots showing bright field (BF) and LM25 xyloglucan, LM2 AGP, LM11/LM27 heteroxylan, and LM1 extensin epitopes. Micrographs representative of at least three replicates. Bar = 1 mm.

To study exudate polysaccharide epitope occurrence at root and root hair surfaces, whole mount preparations of WT and *brb* roots were immunolabelled with the MAbs (Figure 8). While the LM25 xyloglucan epitope was detected over the entire root surface in both genotypes (and root hairs of WT roots), the LM2 AGP and LM11/LM27 heteroxylan epitopes were predominantly at root hair surfaces and detected at very low levels at the surface of root hairless *brb* roots. Intriguingly, the LM1 extensin epitope, abundantly detected in the exudates, was not detected at root surfaces of either genotype. This may indicate that it is a component of a highly soluble polymer or that the epitope is labile during sample fixation, preparation, and immunofluorescence labeling procedures. These observations support the analyses of exudate material and indicate that AGP and heteroxylan epitopes occur abundantly at root hair surfaces from where exudates are released.

### A soil-binding polysaccharide complex is less abundant in *brb* exudates

**Figure 9:**
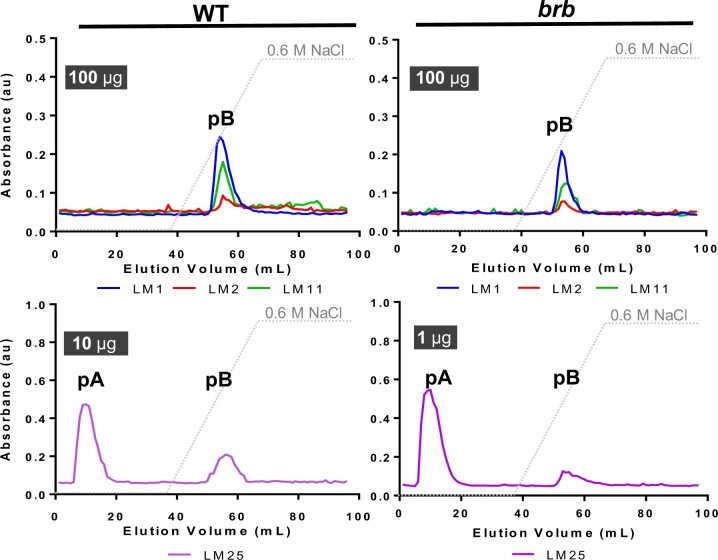
Glycan epitope profiling of anion-exchange chromatographic (AEC) fractionation of WT and *brb* barley HMW root exudates. Varied amounts of HMW root exudates (as shown) were injected in AEC columns and collected in 96 × 1 mL fractions that were analyzed by ELISA. All four epitopes analyzed (LM1 extensin, LM2 AGP, LM11 heteroxylan, and LM25 xyloglucan co-eluted and were present in an acidic peak (pB) indicative of a polysaccharide complex. The LM25 epitope was additionally present in neutral fractions (pA) and analysis indicates an increase in pA/pB in *brb* HMW root exudates relative to WT. Note: for LM25 xyloglucan, 10 µg was injected for WT and 1 µg for analysis of the *brb* HMW root exudate. Data shown are a mean of three biological replicates. Dotted lines show AEC salt-elution gradient.

To explore further the biochemistry of polysaccharides in barley root exudates, the available glycan MAbs that bind to exudates were used as detection tools in microscale analyses of root exudate preparations using anion-exchange chromatography (Figure 9). Because of the differential abundances of the glycan epitopes in exudate samples (Figure 4), chromatographic profiles resulting from injection of differing amounts of exudate are shown. Application of 100 µg of HMW exudate from both WT and *brb* to a 1 mL column and elution with a salt gradient resulted in the co-elution of the LM1 extensin, LM2 AGP, and LM11 xylan epitopes between 0.25 and 0.3 M NaCl with lower peak heights for *brb* (Figure 9). In these profiles the LM25 xyloglucan epitope was abundant and resolved into two peaks. When 10 µg of WT exudate was injected, the LM25 epitope was detected in a major neutral peak (pA) eluting before the onset of the salt gradient and in a smaller peak (pB) co-eluting with the other epitopes at elution volumes of 50–60 mL. Analysis of 1 µg *brb* root exudate also resolved the LM25 epitope into two peaks, with pA much larger relative to the salt-eluted pB when compared to the WT profile.

**Figure 10:**
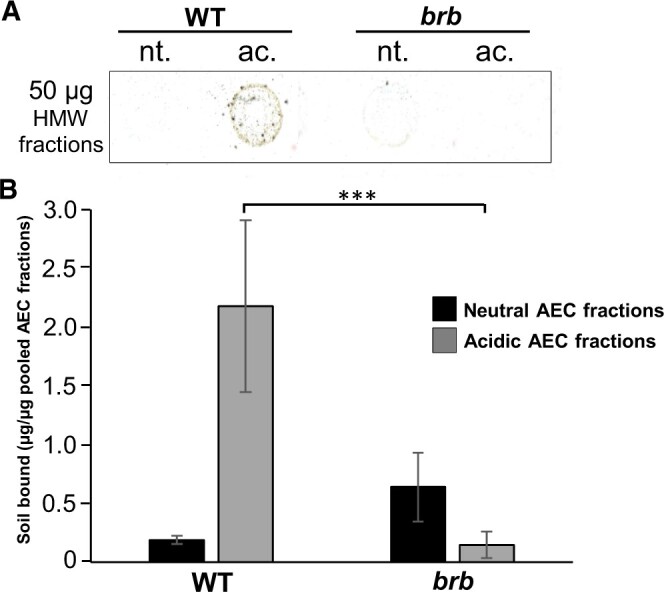
The acidic fraction of WT root exudate has higher soil-binding capacity than the neutral fraction. A, Representative scanned image of soil bound to 50 µg spots of neutral (nt., 1–36 mL) and acidic fractions (ac., 37–72 mL) from anion-exchange chromatography (AEC) analysis of WT and *brb* HMW exudates. B, Quantitation of soil bound to the fractions on a per weight basis analyzed using ImageJ. WT acidic fraction bound more soil than the brb acidic fraction, *P* < 0.001 (*t* test) indicated by three asterisks. *n* = 3, ±SD.

To explore these two regions of the exudate chromatographic profiles further, eluent from fractions 1–36 (neutral) and fractions 37–72 (acidic) from both WT and *brb* were collected after several injections of 1.5 mg material and appropriate fractions pooled, dialyzed and freeze-dried. Equivalent weights of these processed materials were then subject to the nitrocellulose-based soil-binding assay (Figure 10). For WT, the acidic fraction contained greater soil-binding capacity than the neutral fraction on a per weight basis. Low levels of soil-binding were observed for the *brb* pooled exudate fractions and with no difference in soil-binding capacity between the neutral and acidic fractions (Figure 10). While there were no genotypic differences in soil-binding of the neutral fraction, the acidic fraction from WT roots bound ∼10-fold more soil than the acidic fraction from *brb* roots.

## Discussion

This work addresses the molecular factors released by root hairs that contribute to rhizosheath formation in barley. To summarize, root hairs are sites for the secretion of soil-binding polysaccharides of high structural complexity that contain known heteroxylan, xyloglucan, and glycoprotein epitopes. While previous work has highlighted that root hairs physically enmesh soil particles contributing to soil structure and rhizosheath formation (Koebernick et al., 2017; Rabbi et al., 2018), the altered carbohydrate and glycan epitope profile of the HMW root exudates secreted by the root hairless mutant *brb* indicate that in WT root hair exudate polysaccharides also contribute to rhizosheath development.

### Disentangling root hairs and adhesive factors

The role of root hairs in rhizosheath development is well documented, even if the effects of root hair density and length vary between species (Haling et al., 2013; Brown et al., 2017; Pang et al., 2017). Although not all root hairless mutants have altered root architectures relative to WT (Lippold et al., 2021), the *brb* barley genotype morphologically compensated for its lack of root hairs by having greater root mass (Dodd and Diatloff, 2016) and length (Burak et al., 2021; Figure 1B here) than WT. In the case of *brb*, rhizosheath development was still limited with approximately 5-fold less rhizosheath mass per unit root length (Figure 1C). This difference is not entirely due to the physical effects of root hairs enmeshing soil particles, since polysaccharide exudation also substantially differs between the genotypes, with the HMW fraction of *brb* root exudate less able to bind soil in an *in vitro* assay (Figure 2). In contrast, whole root (unfractionated) exudates from *brb* showed greater soil binding than the WT in the nitrocellulose-based assay (Burak et al., 2021). Since low molecular weight metabolites are also likely to contribute to adhesiveness of exudates and can have differing impacts on soil binding (Naveed et al., 2017), further work is needed to disentangle the contributions of different root exudate fractions to soil binding.

Indeed, the absence of root hairs substantially decreased the occurrence of both AGP and heteroxylan epitopes at both the root surface and in released exudates (Figures 5 and 8). In this case, the absence of root hairs has reduced but not abolished the occurrence of epitopes that are abundant at root hair surfaces. Carbohydrate analyses of exudates collected from hydroponates do not differ substantially between genotypes but do indicate a great complexity of structures released by barley roots, of which the available antibodies can only partially resolve. Soil-binding assays and chromatographic evidence indicate a polysaccharide complex (previously suggested for wheat root exudates by sandwich ELISAs (Galloway et al., 2020)) with soil-binding properties that is much decreased within exudates in the absence of root hairs. Both the presence of root hairs and also the chemistry of root hair surfaces/secretions are therefore important factors in rhizosheath formation. Analysis of Arabidopsis root hair adhesiveness also indicates that the chemical nature of root hair surfaces can vary and be under genetic control (De Baets et al., 2020; Eldridge et al., 2021), possibly through varied presentation of carbohydrate structures. An important facet of future work would be to disentangle the roles of polysaccharides at the surface of root hairs, that are likely crucial for the attachment of soil particles, and the roles of equivalent or indeed different sets of polysaccharides that are released from the root hairs into the soil.

### The absence of root hairs and the elevation of xyloglucan in root exudates

Xyloglucan is a much-studied cell wall polysaccharide and is a major component of many, but not all, land plants where its interactions with cellulose microfibrils contribute to the load-bearing properties of primary cell walls (Cosgrove, 2018). It has previously been detected in root tip mucilage (Ropitaux et al., 2019). Although of low abundance in cereal cell walls, the detection of xyloglucan at cereal root surfaces and in root exudates is indicative of specific functions in rhizospheres and rhizosheaths. Unlike root-hair-associated epitopes of AGPs/heteroxylans, greater detection of the LM25 xyloglucan epitope in root-hairless *brb* exudates suggest a compensatory effect in root exudation, brought about by the absence of root hairs. The root surface can be broadly split into three zones for exudate release: root hairs, non-root-hair cells of root axes, and root apices. Immunofluorescence analysis of intact roots indicated equivalent occurrence of the LM25 xyloglucan epitope at the surface of all three zones (Figure 8, Supplemental Figure S1). Immunoprints detecting released polymers from young seedlings (Figure 5) suggested an increased release of xyloglucan from root apices which was more apparent in older plants (Figure 7) but release of this epitope from root axes is also a major factor. Overall, these observations indicate a potential for both the differential and coordinated polysaccharide exudation across the different regions of root surfaces.

The elevated levels of detected xyloglucan in the *brb* exudate preparations are not associated with an enhanced capacity for soil binding. As xyloglucan has been proposed as a soil-binding factor (Galloway et al., 2018), this apparent conflict may be explained by the chromatographic analyses of root exudates. These analyses have indicated two molecular species that carry the LM25 xyloglucan epitope (Figure 9). One of these eluted immediately from an anion-exchange chromatographic column and one required salt for elution and moreover co-eluted with heteroxylan/AGP epitopes. The early-eluting, neutral form of xyloglucan was greatly elevated in the absence of root hairs. We propose therefore that the elevated levels of xyloglucan arise predominantly from root apices and are a distinct molecular form and possibly with distinct functions. In contrast, the later eluting acidic factor, which carries the same xyloglucan epitope in addition to glycoprotein/heteroxylan epitopes, is reduced in *brb* exudates relative to WT.

### A root-hair-released, soil-binding polysaccharide complex

The putative polysaccharide complex that is reduced in *brb* exudates (relative to WT exudates) and that carries xyloglucan, heteroxylan, and glycoprotein epitopes are predominantly associated with root hairs. The presence of the LM2 AGP epitope suggests this class of proteoglycan may act as an organizing factor as has been proposed for other polysaccharide complexes (see Galloway et al., 2020). In addition to potential release in exudates, AGPs have been studied at barley root hair surfaces and implicated in developmental events and interplay with soil microbes (Marzec et al., 2015; Nguema-Ona et al., 2013; Hromadová et al., 2021). Related extensin glycoproteins are also widely detected in root secretions and implicated in root-microbe interactions (Castilleux et al., 2018). This root-hair-associated polysaccharide complex is of an as yet unknown structure, but may contain diverse glucosyl and other glycosyl residues identified in the carbohydrate analyses of the bulk exudates. This polymer will be an important target for further analysis; not only from the perspective of the structure-function relations of an HMW root hair soil-binding factor but also as potentially containing carbohydrate structures distinct from those of cereal cell walls. In addition, understanding the relations between the structural and functional properties of exudate molecules that carry epitopes such as that of LM2 AGP, with those molecules that present the same epitopes at root hair surfaces, is a major knowledge gap. The ability to track exudate polysaccharides through specific epitopes encompassing exudate glycan structures will also be interest to elucidate their origin from roots and function in rhizosheaths and also in wider rhizosphere processes. It will be of interest in future work to determine the specific root regions and indeed cell types responsible for both the presentation and the release of polysaccharides. In such studies, insights from a detailed comparative analysis of hydroponically-grown and solid media/soil-grown roots are likely to be valuable.

In summary, we provide evidence that barley root hairs release a polysaccharide complex containing a range of glycan epitopes that has adhesive properties and this functions in rhizosheath formation. Moreover, this work demonstrates differential release of polysaccharides from barley roots in response to the presence or absence of root hairs and we propose the root apex can modulate its release of xyloglucan when root hairs are absent. The functions of these distinct polysaccharides may be multifarious and some aspects may relate to action as substrates for soil microbes. Bacterial diversity surrounding the roots of another barley root-hairless mutant (*rhl1.a*) was lower than that of the WT, suggesting that root hairs were secreting carbon, thus providing an energy source accessible to the bacteria (Robertson-Albertyn et al., 2017). Through acting as substrates, root exudate polysaccharides may enhance bacterial exopolysaccharide production, thereby further contributing to soil cohesion in rhizosheaths. Additionally, this work highlights further the surprising occurrence of xyloglucan in cereal root secretions and its capacity to be greatly increased in the absence of root hairs, indicating as yet undetermined rhizosphere functions.

## Materials and methods

### Rhizosheath analysis in soil-grown plants

Seeds of WT barley (*Hordeum vulgare* L. cv. Pallas) and *brb* (Gahoonia et al., 2001) were randomly assigned into 4 L pots and planted, at a depth of ∼1 cm, directly into a clay loam soil, two per pot. The pots were then covered with foil until shoots emerged. At this point, ∼4 d after planting, the seedlings were thinned to leave only one shoot per pot. The *brb* and WT pots were randomly distributed in the greenhouse and re-randomized regularly. Both genotypes were germinated and grown in a naturally lit glasshouse with supplementary lighting (photoperiod of 15 h supplying a PPFD of 330 µmol m^−2^ s^−1^ at bench height) and a mean day and night temperature of 22°C and 16°C, respectively. The plants were well-watered every 2 d. This experiment comprised 42 plants, 21 per genotype. Seven plants of each genotype were harvested over three consecutive weeks; 12, 19, and 26 d after planting. At harvest, the plants were systematically removed from the soil, leaving the rhizosheath intact. Each plant was then placed in a metal dish where the rhizosheath soil was washed from the root. The metal trays were then dried at 105°C until they achieved a constant weight; at which point the rhizosheath weight was recorded. The root material was kept in a small amount of water and stored in a fridge for no more than 5 d. Root length was obtained using WinRHIZO (2013e, Regent Instruments Inc., Canada) and a flatbed scanner. The roots were spread out in a clear plastic tray with a thin film of water to keep them separated and scanned at 400 DPI. The scanned images were in 8-bit grayscale and saved in .tiff format. These images were then analyzed in WinRHIZO with a filter excluding any debris with a width:length ratio less than 5.

### Plant hydroponic culture

Barley (*Hordeum vulgare* L. cv. Pallas and *brb*) seeds were germinated on moist filter paper in a Sanyo growth cabinet (MLR-352-PE; Sanyo, Japan) for 7 d with a photoperiod of 16 h, a temperature of 22°C and a mean PPFD of 691 µmol m^−2^ s^−1^. Seedlings were then placed in a mixture of vermiculite and perlite (50:50). Plants were watered every 2 d using dH_2_O. After 7 d of growth, seedlings were placed into a naturally lit glasshouse overnight to acclimatize prior to instituting hydroponic culture as previously outlined (Akhtar et al., 2018). Twelve plants were grown in each 9 L bucket to form one biological replicate. The plants were grown hydroponically using half-strength Hoagland’s nutrient solution (Sigma-Aldrich; H2395-10L, UK) in deionized water for a further 14 d, until harvesting. The glasshouse had a photoperiod of 16 h with a constant temperature of 22°C, with PPFD ranging from 300 to 450 µmol m^−1^ s^−1^. HMW components of hydroponates were concentrated using an ultrafiltration system that had a 30 KDa cut-off membrane. The resulting HMW materials were dialyzed and freeze-dried as detailed (Akhtar et al., 2018; Galloway et al., 2020).

### Soil adhesion assay

Isolated HMW hydroponates of barley genotypes were dissolved into dH_2_O using a starting concentration of 50 µg/5 µL which was then titrated by 1:5 until 0.016 µg/5 µL. A final spot of 5 µL (dH_2_O) was included at the bottom of the nitrocellulose sheet as a negative control. All spots were placed within the center of 1 cm^2^ marked squares and were incubated for 2 h prior to developing the soil adhesion assay, as previously described (Akhtar et al., 2018). The scanning program was as follows for each blot: resolution set at 1,200 dpi, tone curve input 181/output 199. A calibration curve based on gum tragacanth (from *Astragalus* spp; Sigma-Aldrich G1128) and xyloglucan (tamarind seed xyloglucan; Megazyme P-XYGLN) were then used to convert the mean gray values to amount of soil adhered to nitrocellulose as described (Akhtar et al., 2018).

### Monosaccharide linkage analyses, quantitative immunochemical assays, and anion-exchange chromatography of root exudates

Carbohydrate monosaccharide linkage protocols, used for the analysis of barley genotype root HMW hydroponate polysaccharides, were performed by the analytical services of the Complex Carbohydrate Research Center, Georgia, USA as described previously (Galloway et al., 2020). The HMW hydroponates preparations were screened by ELISA with a range of MAbs to glycan epitopes as outlined (Galloway et al., 2020). In some cases, direct assessment by ELISA of the unprocessed hydroponates (before ultrafiltration, dialysis, and freeze-drying) was carried out. For anion-exchange chromatography with epitope detection by ELISA (Cornuault et al., 2014), samples ranging from 1 to 100 µg of freeze-dried HMW compounds were dissolved in 1 mL of 20 mM sodium acetate buffer (pH 4.5) and eluted through a 1 mL HiTrap ANX FF column (GE Healthcare, 17-5162-01). Samples were eluted at a flow rate of 1 mL/min with 20 mM sodium acetate buffer pH 4.5 for 36 mL, then a linear gradient of 0–100% (v/v) 0.6 M NaCl (in 50 mM sodium acetate buffer pH 4.5) until 70 mL, followed by 100% 0.6 M NaCl for another 26 mL. In total, 96 (1 mL) fractions were collected. These were analyzed by ELISA as described (Cornuault et al., 2014). In certain cases, fractions from 1 to 36 and from 37 to 72 were collected, pooled, and dialyzed against dH_2_O using a 3.5 KDa cut-off point Spectra/pro membrane (Spectrumlabs, US) and then freeze-dried. The resulting materials were then used in the soil adhesion assay.

### MAbs and immunoprinting/immunoprofiling procedures with intact seedlings

The panel of MAbs used to survey exudate preparations has been described elsewhere (Galloway et al., 2020). MAbs used for the analysis of exudates from whole barley seedlings in root prints, seedling root exudate collection experiments and immunofluorescence analyses include LM1 extensin (Smallwood et al., 1995), LM2 AGP (Smallwood et al., 1996), LM11 heteroxylan (Ruprecht et al., 2017), LM27 heteroxylan (Cornuault et al., 2015), and LM25 xyloglucan (Pedersen et al., 2012). Immunoprinting on nitrocellulose substrates (Perkin Elmer Protran nitrocellulose membrane, 0.45 µm) was carried out as described (Galloway et al., 2020). In some cases, young seedlings were placed on pieces of nitrocellulose. With the larger root systems of plants that had grown in hydroponic systems for ∼2 weeks, root systems were arranged on Perspex 20 cm × 20 cm trays, and then moist nitrocellulose sheets (15 cm × 15 cm) were placed on the roots for 10 min before removal of sheets for processing. In some cases, the roots and in-contact nitrocellulose sheets were inverted so that root systems rested on the sheets and then incubated for 30 min. For exudate analysis from specific root regions, 7- to 8-d-old seedlings were placed in a humid chamber and 0.5–1 cm of root tip incubated in 1.5 mL water, and a region of root body (3–5 cm in length) separated from root tip by 2 cm was incubated in 5.5 mL water for 2 h. In the cases of WT seedlings root hairs would be presented in both the tip and axis regions (see Supplemental Figure S1). The seedlings were then removed and the exudate water samples were assessed directly by ELISA as described (Galloway et al., 2020).

### Immunofluorescence labeling of barley root surfaces

To observe carbohydrate epitopes at root surfaces, 1 to 2 cm regions of whole barley roots with root hairs (WT) or equivalent regions of *brb* roots were excised and placed overnight in a 4% (w/v) formaldehyde fixative and processed for whole mount immunofluorescence labeling procedures essentially as described (Jackson et al., 2012). After antibody incubations, intact root regions were mounted using Citifluor AF2 antifade reagent (glycerol suspension; Agar Scientific Stansted, U.K.) and examined using an Olympus BX-61 microscope with epifluorescence irradiation from a mercury lamp and with excitation (∼480 nm) and emission (∼510 nm) for fluorescein isothiocyanate (FITC) fluorescence detection. Images were captured using a Hamamatsu ORCA285 camera and Volocity software. For each antibody, a manual exposure time was maintained for the capture of all shown micrographs across the two barley genotypes.

### Statistical analysis

For soil-grown plants, two-way ANOVA resolved the effects of genotype, time of harvest, and their interaction on rhizosheath weight and root length, with means discriminated using Student’s *t* test. Subsequently, ANCOVA was used to determine genotypic differences in rhizosheath weight per unit root length. In the case of seedling root tip/root axes incubation experiments, a two-factor ANOVA resolved the effect of genotype and root position. Other comparisons of WT and *brb* exudates (soil binding capacity monosaccharide linkage analysis and glycan epitope mapping) utilized Student’s *t* test. Differences were considered significant when the *P*-values were below 0.05.

## Supplemental data

The following materials are available in the online version of this article.

**Supplemental Figure S1.** Whole-mount immunofluorescence analysis of the LM25 xyloglucan epitope at the surface of root apices of barley WT and *brb*.

**Supplemental Table S1.** Monosaccharide linkage analysis of barley WT and *brb* HMW hydroponate samples.

## Supplementary Material

kiac341_Supplementary_Data

## Notes

A.F.G., J.A., E.B., K.J.F., I.C.D., and P.K. designed the research; E.B. carried out rhizosheath analyses; A.F.G., J.A., and E.B. carried out the isolation, soil-binding studies, and immunoanalyses of root exudates: S.E.M, J.A., and P.K carried out seedling prints and immunofluorescence studies. A.F.G and P.K. drafted the manuscript. All authors contributed to and approved the final version of the article.

The author responsible for distribution of materials integral to the findings presented in this article in accordance with the policy described in the Instructions for Authors (https://academic.oup.com/plphys/pages/general-instructions) is: Paul Knox (j.p.knox@leeds.ac.uk).
