# Supplementary material for: Altered properties and structures of root exudate polysaccharides in a root hairless mutant of barley
Source: Plant Physiol. 2022 Jul 25;190(2):1214–27. doi: 10.1093/plphys/kiac341 (PMC9516773; doi:10.1093/plphys/kiac341)

Supplemental data for *Galloway et al*. **Altered properties and structures of root exudate polysaccharides in a root hairless mutant of barley**

Supplemental Table S1. Monosaccharide linkage analysis of barley WT and *brb* HMW hydroponate samples. Linkages shown as Mol%. Combined data (n = 4) showing means and SD from hydroponates grown on two separate occasions. Column headed with **Sig.** indicates significantly different means between the two genotypes (*P < 0.05, **P < 0.01, t-test). Underlines data indicate mean values for the total of each monosaccharide shown in bold.

		**WT**		***brb***		
		**Mean**	**SD**	**Mean**	**SD**	**Sig.**
Ara*p*	Terminal	2.50	1.85	2.75	2.05	
	2-	0.05	0.05	0.13	0.13	
	4-	0.85	0.36	0.98	0.42	
Ara*f*	Terminal	10.93	8.01	11.80	8.26	
	2-	1.28	1.28	0.95	0.95	
	3-	0.45	0.45	0.33	0.33	
**Arabinose**	**Total**	16.05	8.36	16.93	7.92	
Fuc	Terminal	0.25	0.26	0.10	0.10	
	3-	0.13	0.13	0.10	0.10	
**Fucose**	**Total**	0.38	0.39	0.20	0.20	
Gal*p*	Terminal	4.00	1.72	1.65	0.35	*
	3-	1.95	1.57	0.85	0.60	
	4-	0.10	0.10	0.08	0.08	
	6-	0.68	0.08	0.38	0.08	**
	3,6-	0.93	0.93	0.25	0.26	
Gal*f*	Terminal	0.60	0.62	0.25	0.25	
**Galactose**	**Total**	8.25	0.52	3.45	0.42	***
Glc	Terminal	4.58	2.60	2.70	1.81	
	2-	0.38	0.13	0.20	0.00	*
	3-	0.33	0.08	0.18	0.04	*
	4-	19.48	7.81	22.73	9.88	
	6-	0.73	0.48	0.83	0.63	
	2,4-	0.23	0.15	0.30	0.07	
	3,4-	0.40	0.40	0.70	0.00	
	4,6-	17.20	7.13	20.10	7.56	
	2,4,6-	0.68	0.68	1.53	1.53	
	3,4,6-	6.05	6.07	6.35	6.35	
**Glucose**	**Total**	50.03	10.78	55.25	11.64	
Xyl	Terminal	14.03	3.77	16.90	3.27	
	2-	0.55	0.21	0.73	0.33	
	4-	0.88	0.33	1.73	0.48	*
	2.4-	0.15	0.15	0.20	0.20	
	3,4-	0.43	0.43	0.20	0.20	
**Xylose**	**Total**	16.03	4.86	19.75	4.45	
Rha	Terminal	1.48	0.84	0.50	0.16	*
	2-	0.25	0.26	0.13	0.13	
	3-	1.18	1.18	0.38	0.38	
	2,3-	0.35	0.36	0.10	0.10	
**Rhamnose**	**Total**	3.25	0.50	1.10	0.21	***
GlcA	Terminal	1.23	1.24	0.43	0.43	
**Glucuronic Acid**	**Total**	5.83	3.43	2.63	0.15	
GalA	terminal	0.23	0.23	0.13	0.13	
	4-	0.05	0.05	0.05	0.05	
**Galacturonic Acid**	**Total**	0.28	0.28	0.18	0.18	
Man	Terminal	1.80	1.81	0.83	0.83	
	2-	1.30	0.56	0.68	0.18	
	3-	0.13	0.13	0.05	0.05	
	4-	0.38	0.11	0.23	0.11	
	6-	0.05	0.09	0.08	0.08	
	2,3-	0.85	0.85	0.65	0.65	
**Mannose**	**Total**	4.50	1.49	2.50	0.45	*

Supplemental Figure S1. Whole mount immunofluorescence analysis of the LM25 xyloglucan epitope at the surface of root apices of barley WT and *brb*. The epitope is present at all root surfaces. Bar = 0.5 mm.

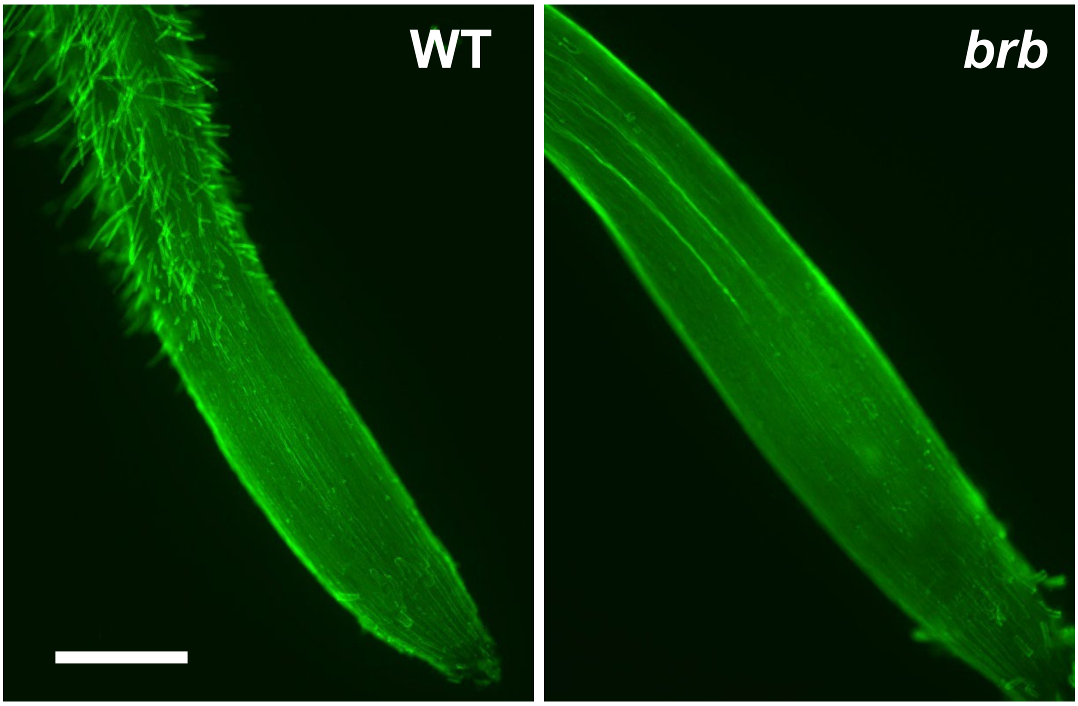

Supplement: kiac341_Supplementary_Data [file kiac341_supplementary_data.pdf]
